# Performance of Cell-Free DNA Screening for Fetal Common Aneuploidies and Sex Chromosomal Abnormalities: A Prospective Study from a Less Developed Autonomous Region in Mainland China

**DOI:** 10.3390/genes12040478

**Published:** 2021-03-25

**Authors:** Yunli Lai, Xiaofan Zhu, Sheng He, Zirui Dong, Yanqing Tang, Fuben Xu, Yun Chen, Lintao Meng, Yuli Tao, Shang Yi, Jiasun Su, Hongqian Huang, Jingsi Luo, Tak Yeung Leung, Hongwei Wei

**Affiliations:** 1 Birth Defects Prevention and Control Institute of Guangxi Zhuang Autonomous Region, Nanning 530000, China; laiyunlilyl@163.com (Y.L.); heshengbio@163.com (S.H.); tangyanqing2006@126.com (Y.T.); fbxu1513@126.com (F.X.); ych_yun@outlook.com (Y.C.); menglt_fy2017@163.com (L.M.); taoyuli520@163.com (Y.T.); yishang@aliyun.com (S.Y.); sujiasun@126.com (J.S.); money3838110@sina.com (H.H.); ljs0815freedom@163.com (J.L.); 2 Genetic and Metabolic Central Laboratory, Maternal and Child Health Hospital of Guangxi Zhuang Autonomous Region, Nanning 530000, China; 3 Guangxi Clinical Research Center for Fetal Diseases, Nanning 530000, China; 4 Department of Obstetrics and Gynaecology, The Chinese University of Hong Kong, Hong Kong, China; zhuxf@link.cuhk.edu.hk (X.Z.); elvisdong@cuhk.edu.hk (Z.D.); tyleung@cuhk.edu.hk (T.Y.L.); 5 Genetics and Prenatal Diagnosis Center, The First Affiliation Hospital of Zhengzhou University, Zhengzhou 450052, China; 6 Shenzhen Research Institute, The Chinese University of Hong Kong, Shenzhen 518000, China; 7 The Chinese University of Hong Kong-Baylor College of Medicine Joint Center for Medical Genetics, Hong Kong, China; 8 Department of Obstetrics and Gynaecology, Maternal and Child Health Hospital of Guangxi Zhuang Autonomous Region, Nanning 530000, China

**Keywords:** noninvasive prenatal screening, cell-free DNA, common aneuploidies, rare chromosomal abnormalities, less developed region, follow-up information

## Abstract

To evaluate the performance of noninvasive prenatal screening (NIPS) in the detection of common aneuploidies in a population-based study, a total of 86,262 single pregnancies referred for NIPS were prospectively recruited. Among 86,193 pregnancies with reportable results, follow-up was successfully conducted in 1160 fetuses reported with a high-risk result by NIPS and 82,511 cases (95.7%) with a low-risk result. The screen-positive rate (SPR) of common aneuploidies and sex chromosome abnormalities (SCAs) provided by NIPS were 0.7% (586/83,671) and 0.6% (505/83,671), respectively. The positive predictive values (PPVs) for Trisomy 21, Trisomy 18, Trisomy 13 and SCAs were calculated as 89.7%, 84.0%, 52.6% and 38.0%, respectively. In addition, less rare chromosomal abnormalities, including copy number variants (CNVs), were detected, compared with those reported by NIPS with higher read-depth. Among these rare abnormalities, only 23.2% (13/56) were confirmed by prenatal diagnosis. In total, four common trisomy cases were found to be false negative, resulting in a rate of 0.48/10,000 (4/83,671). In summary, this study conducted in an underdeveloped region with limited support for the new technology development and lack of cost-effective prenatal testing demonstrates the importance of implementing routine aneuploidy screening in the public sector for providing early detection and precise prognostic information.

## 1. Introduction

Noninvasive prenatal screening (NIPS) using circulating cell-free DNA (cf-DNA) in maternal plasma has been widely implemented as a routine screening method for fetal chromosomal aneuploidy in obstetric practice. Previous studies have indicated that NIPS showed a high accuracy in detecting common trisomies (Trisomy 21, Trisomy T18 and Trisomy T13) and the feasibility in reporting sex chromosome abnormalities (SCAs) by using massively parallel sequencing (MPS), chromosome-specific sequencing (CSS) and single nucleotide polymorphism (SNP)-based methods via various next-generation sequencing (NGS) platforms [1,2,3,4]. Currently, it is recommended for aneuploidy screening in all pregnancies, regardless of maternal age or other risk factors [5], by the American College of Obstetricians and Gynecologists (ACOG).

Compared with common aneuploidies, the prevalence of other rare chromosomal abnormalities diagnosed before the age of one year old was 7.4/10,000 births [6]. NIPS sequenced at a read-depth of ~0.04–0.15× for common fetal trisomies and SCAs were named standard NIPS. In comparison, expanded NIPS refers to NIPS sequenced at a read-depth of ~0.15–0.3× and expanded the detection scope to additional chromosomal abnormalities (such as microdeletion/duplication syndrome) and rare autosomal trisomies (RATs) [7,8,9,10]. However, the performance varies among variable sequencing read-depths, as well as methods applied (counting or SNPs approach). The positive rate of such abnormalities by expanded NIPS ranged from 0.12 to 1.58% among all submitted cases [7,8,10,11,12,13], with the positive predictive values (PPVs) for copy number variants (CNVs), RATs and other abnormalities as 28.99–57.14% [8,10,12,13], 6–58.82% [8,10,13,14] and 0–64% [10,11,12], respectively. Although expanded NIPS is able to provide an increased yield of the overall chromosomal abnormalities [8], it is still difficult to evaluate its sensitivity and specificity due to the differences exist in (1) types and spectrum of chromosomal abnormalities, (2) resolution (sizes) [13,15,16], (3) sequencing platforms [8,17], (4) sequencing parameters analyzed (such as sequencing read-depth and cf-DNA%) [17,18] and (5) referral indications [12,16,19,20]. In addition, as the incidence of individual aberration is low, validation in a cohort with large sample size with pregnancy outcomes is challenging but still needed. 

In China, NIPS has been recommended as a routine prenatal screening test to assess the risks associated with pregnancies [5]. Besides commercial companies, hospitals accredited for prenatal diagnosis have started offering standard NIPS, while expanded NIPS also becomes widely used in some developed areas of China [21]. However, most of the published data from China were collected from different centers. Due to the small sample size without follow-up data, the number of different types of abnormalities from each single center was insufficient for comparison [8,10,12], or represent the general population. Moreover, the utility of expanded NIPS in the general obstetric population is still on debate. Therefore, evaluating the performance and utility of standard NIPS in a prospective large sample size from a local center is necessary and urgent. We used less developed Guangxi Zhuang Autonomous Region in China as an example. For this study, we included Zhuang, the second largest ethnic group in China, and other minorities’ groups. Guangxi region has a large genetic heterogeneity and a high incidence of birth defects. In perinatal period, the incidences of birth defects are about 156/10,000 births in Guangxi (Guangxi Birth Defect Prevention Report, 2020). Additionally, the expanded NIPS currently offered in Guangxi region is very expensive due to its higher sequencing read-depth (~0.15–0.3×; reagent cost as USD120 per case), compared with standard NIPS (~0.04–0.15×, reagent cost as USD60 per case). As shown in previous studies published in Chinese cohorts, a total of 1.2% fetal chromosome abnormalities were detected by NIPS [8] and 16.7–19.0% fetal chromosome abnormalities were diagnosed by chromosomal microarray analysis (CMA) and low-pass genome sequencing [8,22]. This demands a cost-effective approach, such as standard NIPS, for prenatal screening, mainly to detect common fetal aneuploidies.

Herein, it is a prospective study about the performance of screening for common aneuploidies and sex chromosomal abnormalities, using noninvasive assessment of the fetal genome from maternal serum-noninvasive prenatal screening test in Guangxi Zhuang Autonomous Region, in China. A large sample size of 86,262 consecutive pregnant women who received standard NIPS in a local single center were recruited for evaluating the performance of detecting different chromosomal abnormalities in different risk cohorts. In addition, outcome follow-up was successfully conducted in the majority of the cases (*n* = 83,671, 97%). Overall, this study provided valuable references for genetic counseling and clinical application. Eventually, its implementation could significantly reduce the rate of birth defects in this region.

## 2. Materials and Methods 

### 2.1. Study Population and Samples

This research was approved by the ethics review committee of the Maternity and Child Health Hospital of Guangxi Zhuang Autonomous Region, in accordance with the World Medical Association Declaration of Helsinki: Ethical Principles for Medical Research Involving Human Subjects. Consecutive pregnant women were referred for NIPS at the Center Laboratory of Genetic and Metabolic Department, Maternity and Child Health Hospital of Guangxi Zhuang Autonomous Region from May 2015 to December 2018. All patients were recruited with the following criteria, based on the guidelines of the American College of Medical Genetics and Genomics (ACMG) [23] and the National Health Commission of the People’s Republic of China (2016): maternal age ≥16, singleton pregnancy ≥12 weeks of gestation, and no history of transfusion or transplantation during past years.

**Figure 1:**
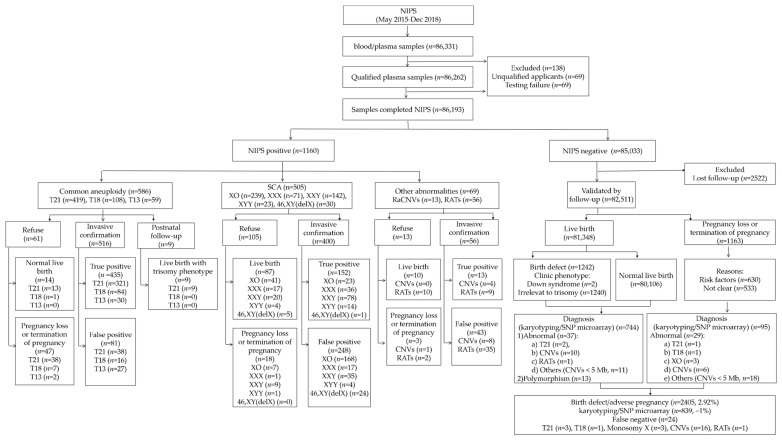
Flowchart of noninvasive prenatal screening (NIPS) and outcomes of singleton pregnancies in a single center.

According to prenatal screening protocols in the hospital: Ultrasound examinations were provided routinely for all pregnant women in the obstetric checkpoints (11–13^+6^ weeks, 16–18 weeks, 22–24 weeks, 28–32 weeks and after 38 weeks) to measure and assess for fetal nuchal translucency (NT), structural abnormalities and growth and development. Apart from ultrasound examinations, (1) for first-trimester Down syndrome screening (DSS1) (11–13^+6^ weeks), detection of pregnancy-associated plasma protein A and free β-chorionic gonadotrophin were offered; and (2) for second-trimester DSS (DSS2, 15–20^+6^ weeks), detection of α-fetoprotein, unconjugated estriol and free β-chorionic gonadotrophin were provided. For women with intermediate/high-risk pregnancy indicated by either first- or second-trimester screening, NIPS was offered based on the guidelines of the National Health Commission of the People’s Republic of China (2016). Those with structural abnormalities reported by ultrasound screening include cardiac malformations, cleft lip and palate, fetal hydrops, limb malformations, cystic hygroma, renal dysplasia, lung cystadenomas, etc. They are excluded in this study, resulting in a total of 86,262 pregnancies. All eligible pregnant women undertook pre-test counseling and provided written informed consents. A flowchart of this study is illustrated in Figure 1.

For each participant, 10 mL maternal peripheral blood was collected with Streck Cell-Free DNA BCT tubes (La Vista, New England, USA) and stored at room temperature (around 24 °C). Each sample was then proceeded (within 72 h after collection) for a two-step centrifugation, centrifuged at 1600× *g* for 15 min (4 °C) and 16,000× *g* for 10 min (4 °C), to isolate the supernatant plasma. Subsequently, the plasma sample was transferred to a 1.5 mL fresh EP tube, stored at −20 °C for the first day and at −80 °C afterwards, for further processing. Each plasma sample was frozen and thawed only once, to avoid DNA degradation. 

### 2.2. DNA Extraction, Library Construction, Sequencing and Bioinformatics Analysis

NIPS was performed by following the manufacturer’s instructions. Briefly, cf-DNA was extracted from 1.2 mL plasma, using magnetic beads-based plasma cf-DNA extraction kit (Berry Genomics, Beijing, China), subjected to library construction and purification, using the magnetic beads-based MPS DNA library construction and purify kit (Berry Genomics, Beijing, China). Libraries were quantified by Kapa SYBR fast qPCR kit (Kapa Biosystems, Wilmington, USA), on a StepOnePlus platform (ABI, Thermo Fisher Scientific, Waltham, MA, USA). Libraries of 96 samples with barcodes were then pooled together with equimolar and subjected for single-end sequencing (37 base-pairs with another 8 base-pairs as index) on a Nextseq-500 platform (Illumina, San Diego, CA, USA).

Sequencing reads were endured if sequencing quality value (Q30) was >85%, and GC content ranged from 38 to 42. Overall, after mapping to the human reference genome (GRCh37/hg19) by RUPA software developed by Berry Genomics [8], a minimum of 3 million uniquely aligned reads was obtained for each sample. The data were then processed by a Bambni Test Data Analysis System 3.7 (Berry), with parameters reported in previously published studies [8,24]. After GC correction, fetal fraction (FF) was estimated by using elastic net (ENET) algorithm [25]. Subsequently, chromosome aneuploidy was reported using the criteria of Z-score ≥ 3 (trisomy) or ≤ −3 (monosomy). When different fetal fractions were reported by two algorithms (ENET and chromosome Y-based), mosaic chromosome aneuploidy was considered. The analytical algorithm for CNVs was reported in previous studies [8,26], with a resolution of 5 Mb.

All samples failed in any of the following steps were requested for resubmission: (1) samples were requested for resampling (such as whole blood or plasma with poor quality, low FF, multiple failing factors of sequencing and failure in re-experiment) and processed by using the peripheral blood/plasma procedure, as described above; (2) samples were requested for re-examining/re-experiment due to failed quality control (QC), such as high or low concentration of DNA extraction, abnormal peak or low concentration of library construction, GC content bias, insufficient data amount, multiple chromosome abnormalities (Multis), borderline Z-score, multiple failing factors of sequencing, etc. A repeat of library construction, sequencing and data analysis was performed afterwards; the same procedure (DNA extraction, library construction, sequencing and bioinformatics analysis) was conducted as described above. 

### 2.3. Clinical Outcome and Follow-Ups

Women with positive NIPS results were offered confirmatory diagnostic tests (karyotyping and/or SNP array) via invasive procedure and post-test counselling for pregnancy management. Clinical evaluation was carried out for those pregnancies kept to term birth. Follow-up information was obtained from all NIPS participants by phone or interview, including any ultrasound abnormalities and/or soft markers found during pregnancies, pregnancy outcomes, any dysmorphic features and abnormalities found in newborns.

### 2.4. Data Analysis

Open-source tool python (https://www.python.org/) was used for data plotting and statistical analysis. Mean, standard deviation, median and range were calculated for each of the biographic datum (such as gestational age, maternal age, weight and body mass index (BMI)), as well as FF. Student’s *t*-test was used to compare the differences of FF between reportable results and no-call groups, and between second-trimester and third-trimester groups. Comparisons of FF and related factors (such as gestational age, maternal age, weight, BMI and Z-score) among multiple groups were performed by one-way ANOVA. Chi-Square test and Fisher’s exact test (*n* < 5) was used to (1) compare the prevalence of pregnancy women opted for continuing pregnancies in groups, with or without confirmation by prenatal diagnosis after receiving a positive results by NIPS; (2) compare the prevalence of pregnancy in women who opted for termination of pregnancies (TOPs) in groups between common trisomies and SCAs, with confirmation by prenatal diagnosis after receiving a positive results by NIPS; and (3) analyze the difference of screen-positive rate (SPR) and PPV among different referral indications. A *p*-value < 0.05 was considered as statistically significant. Those no-call samples and low-risk cases without follow-up results were excluded from the study, when calculating the prevalence, sensitivity and specificity, PPV, negative predictive value (NPV), false positive rate (FPR), and false negative rate (FNR) of the test.

## 3. Results

### 3.1. Characteristics of Study Population

A total of 86,262 singleton pregnancies were referred for NIPS testing during the study period, and NIPS yielded results in 86,193 (99.9%) cases. Maternal age ranged from 16 to 54 years, with a median of 33 years. Of the 86,193 cases, 37,387 pregnancies (37,387/86,193, 43.38%) were advanced maternal age (AMA). In addition, the median gestational week was 17^+3^ weeks (ranged 12–38 weeks), while 1456 (1.69%) of pregnant women were with obesity (BMI ≥ 30 kg/m^2^) observed [27]. Furthermore, 3.25% (2802/86,193) of women conceived with assisted reproductive technology (ART). The demographic characteristics of these participants are summarized in Table 1.

### 3.2. Testing Failure Rate and Results Distribution of NIPS

**Figure 2:**
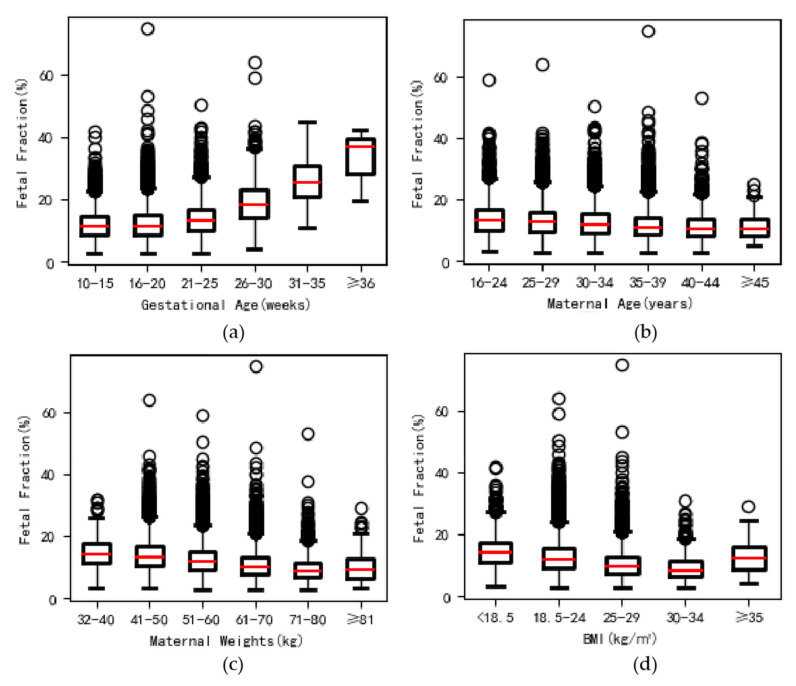
Comparison of fetal fraction with gestational age, maternal age, weight and body mass index (BMI). (**a**) A significant positive relationship between fetal fraction and gestational age (y = 0.342x + 6.555, r = 0.214, *p* < 0.001). (**b**) A significant change in fetal fraction was observed with maternal age (y = −0.146x + 17.420, r = −0.168, *p* < 0.001). (**c**) Fetal fraction decreased gradually with maternal weights (y = −0.447x + 60.764, r = −0.263, *p* < 0.001). (**d**) Fetal fraction decreased gradually with BMI (y = −0.409x + 21.726, r = −0.261, *p* < 0.001).

After sample submission, there were three QC steps; samples that failed in any step were requested for sample resubmission for NIPS test (Table 2): (1) poor quality of blood plasmas (hemolysis, coagulation and lipemia) = 446 samples (0.52%), and all were resampling; (2) failure in DNA extraction and library construction = 1277 samples (1.48%) for repeating and 48 samples for resampling (0.06%); and (3) failure in sequencing and data analysis = 1359 samples (1.58%) for repeating and 303 samples for resampling (0.35%). Overall, NIPS failed in yielding results in 0.08% (69/86,262) of women (Table 2). Among the group with low FF (*n* = 125, mean FF as 2.95%), the average FF of the resubmitted samples was increased to 5.49% (range from 3.19 to 15.53%), and the average FF was significantly higher in the cases with yielded results (5.49%, *n* = 110), as compared to those without (or namely no-call, mean 2.82%, *n* = 15, *p* < 0.001 (Table 2). The FF was significantly increased with gestational weeks (*p* < 0.001) but obviously decreased with maternal weight and BMI (both *p* < 0.001) (Figure 2). We also found significantly higher FF in the second-trimester group (*n* = 78,231, 90.76%, cf-DNA = ~12.57%) than that in the third-trimester (*n* = 584, 0.68%, cf-DNA = ~23.0%,) (*p* < 0.001, not shown in the table). The results also showed a positive relationship between FF and Z-score of trisomy 21 (*p* < 0.001). The detailed results are shown in Table A1.

Among the 86,193 cases (99.9%) with a reportable NIPS result, there were 1160 fetuses reported to be high-risk (1.35%) by NIPS, of which 586 cases (50.52%) were common trisomies, 505 cases (43.53%) were SCAs and 69 cases (5.95%) were rare chromosomal abnormalities. Overall, 84.57% of them (981/1160) received an invasive/clinical diagnosis for further confirmation. 

Furthermore, there were 85,033 fetuses with low-risk results (98.65%) by NIPS, and 97.03% (82,511/85,033) of them were successful follow-ups (with contacted and clinical outcome provided). Further prenatal or postnatal diagnosis was made in approximately 1% of cases (839/82,511), mainly due to ultrasound anomalies (identified post-NIPS) or postnatal phenotypic abnormalities. There were twenty-four cases missed by previous NIPS (low-risk results), including common trisomies (*n* = 4) and monosomy X (*n* = 3), CNVs (≥5 Mb) (*n* = 16) and RATs (*n* = 1) (Table A2). Totally, the validated number of pregnancies used for further analysis was 83,671 (1160 high-risk and 82,511 low-risk NIPS cases).

### 3.3. Performance of NIPS for Detecting Common Aneuploidies

Among the 586 cases with common trisomies, 525 of which underwent invasive confirmation (89.6%). For SCAs, there were 400 cases with diagnostic confirmations (79.2%, 400/505). Thus, the SPR of common aneuploidies and SCAs were 0.7% (586/83,671) and 0.6% (505/83,671) (Table 3). The SPR of common trisomies had significant differences among different referral indications (*p <* 0.001, from highest to lowest): ultrasound soft markers (1.57%), DSS high-risk (1.1%), AMA (0.96%), ARTs (0.81%), maternal anxieties (0.67%), DSS intermediate-risk (0.37%) and others (0.28%). For SCAs, there was no significant difference of SPRs observed among these subgroups (ranged from 0.44% to 0.74%, *p* = 0.16, Table 4).

Among the 525 cases with common trisomies who received invasive confirmations, 444 were confirmed (330 cases of T21, 84 cases of T18 and 30 cases of T13). Among these, 17 cases had atypical cytogenetic findings, including mosaicism of trisomies (mosaic level: 7 to 58%), Robertsonian T21 and other chromosomal structural abnormalities. Therefore, the PPV for T21, T18 and T13 were 89.67%, 84.00% and 52.63%, respectively. By considering those common trisomies (three with T21 and one with T18) identified in NIPS low-risk pregnancies, the combined sensitivity, FPR, PPV and NPV for common trisomies were 99.11%, 0.10% and 84.57% and 99.995%, respectively. In comparison, among the 400 cases with SCAs receiving further confirmations, the combined FPR and FNR were 0.30% and 1.94%, respectively. It resulted in a combined PPV of 38.00%. Among three categories of SCAs, both FPR and FNR were highest for Monosomy X, resulting in a lowest PPV of 12.04%. In comparison, in the group which indicated decrease of X chromosome DNA in male fetuses, the FPR and FNR of 46,XY(delX) were both the lowest among three groups. However, the PPV of 46,XY(delX) was also the lowest, probably owing to the low SPR (0.04%). The detailed results are shown in Table 3.

Overall, NIPS yielded high sensitivity and specificity in detecting common trisomies and SCAs among different referral indications (Table 3). The combined PPV for common aneuploidies and SCAs was 64.43%. For cases with Trisomy 21, the PPVs in cases with AMA, DSS high-risk and ultrasound cohorts were higher than that in the other cohorts (*p* = 0.07), while the PPVs for monosomy X varied in different referral indications (range from 0 to 50%, *p* = 0.06, Table 4); however, no significant differences were found for these PPVs. 

### 3.4. Additional Findings

Apart from common trisomies and SCAs, there were 69 cases with other chromosomal abnormalities reported by NIPS, accounting for 5.95% (69/1160) of the overall positive cases. Thirteen cases were CNVs (≥5 Mb), and 56 cases with RATs. For CNVs, CNV sizes ranged from 6 to 32.5 Mb for deletions (*n* = 8) and 5.5 to 99 Mb for duplications (*n* = 9), respectively. Twelve of these 13 cases (92.3%) underwent invasive diagnosis, and the results confirmed four CNVs (4/12, 33.3%). In addition, among pregnancies that sought invasive prenatal diagnosis in the NIPS low-risk group, there were an additional 16 CNVs (≥5 Mb) identified (Table 3).

For RATs, there were 17 cases with increased dosage of chromosomes (likely trisomy), 15 cases with decreased dosage of chromosomes (likely monosomy) and 24 cases with Multis by NIPS. However, only nine of them (9/44, 20.5%) were confirmed by invasive testing. In addition, an additional RAT (mosaic T22) was reported in NIPS low-risk group (Table A2). For these RATs with Multis (*n* = 24), 75% (18/24), cases were reported to have more than three chromosome abnormalities, and most of them included common trisomies. There were only five cases confirmed by further invasive testing (5/22), and three of them, in fact, were common trisomies. 

In addition, in one case with increased dosage of chromosome 18 by NIPS (Table A2, No. 46), CMA showed uniparental disomy 18, which might be resulted from trisomy rescue. We further followed up those cases with normal fetal karyotypes (*n* = 17), and maternal aplastic anemia and gallstone were identified in two cases, respectively. Interestingly, the diagnostic results presented a high combined PPV (87.5%, 7/8) of segmental deletion in chromosome 18 (chr18-) that was reported in 10 cases with CNVs and RATs (Table A2).

Overall, for CNVs and RATs, including Multis, the PPVs were 33.33% and 20.45%, respectively. The combined PPV, NPV and FPR for these additional abnormalities were 23.21%, 99.98% and 0.05%, respectively. In total, when NIPS detection scope were expanded from common trisomies and SCAs to genome-wide abnormalities (CNVs and RATs), the combined PPV was only slightly decreased from 64.43% to 62.08%, and the FPR was also slightly increased from 0.40 to 0.45% (Table 3), both of which were similar to a previous study [12].

### 3.5. Clinical Outcome and Follow-Up

Among the 82,511 NIPS reported low-risk cases with pregnancy outcomes available, 1242 cases (1.51%) had birth defects and 1163 cases (1.41%) had adverse pregnancy outcomes; most of them (99.0%, 2381/2405) were not affected by the chromosomal abnormalities that were within our study scope (Table A3 and Table A4). Among the 24 false negative cases, four cases with common trisomies (three T21 and one T18) presented abnormal phenotype in their fetal or neonatal periods, and three cases with mosaicism of monosomy X. In addition, antenatal and/or postnatal anomalies were reported among the 12 of the other 17 cases with other chromosomal abnormalities detected (70.59%).

Among the 1160 cases with positive results from NIPS, there were 609 fetuses confirmed with common trisomies, SCAs or the other chromosomal abnormalities by further invasive diagnosis. Follow-up was conducted in 444 (100%) fetuses with common trisomies. Among them, 95.05% (422/444) opted for TOP and 4.95% (22/444) continued the pregnancies, respectively. Pregnancies opted for TOP included common trisomies (95.05%, 422/444), or SCAs (52.63%, 80/152) respectively, showing significant difference between the two groups (*p <* 0.001). 

For 179 cases with NIPS high-risk results but that did not undergo invasive confirmations, follow-up was successfully conducted in all cases. Sixty-eight (38.0%) opted for termination, and 111 (62.0%) continued pregnancies, respectively. In addition, among these 179 cases without invasive confirmations, 82.9% (87/105) of cases reported to have SCAs by NIPS opted for continuing pregnancies, which was significantly higher than that with confirmation of SCAs (47.4%, 72/152, *p <* 0.001; Table A4). 

## 4. Discussion

Our study provides the largest sample size of NIPS pregnancy outcome data (*n* = 83,671) from a single center. This prospective population-based study, demonstrated that standard NIPS is a cost-effective method for detecting fetal common aneuploidies in a less developed region. This is further supported by high quality of pregnancy outcome follow-up data. Among the 86,193 pregnant women received a NIPS result with the follow-up tests, the PPVs for T21, T18, T13 and SCAs provided by standard NIPS were at 89.7%, 84.0%, 52.6% and 38.0%, respectively, which were comparable to those reported by higher sequence read depth expanded NIPS (95%, 82%, 46% and 47%) [8]. The DR, PPV, NPV and FPR of detecting common trisomies were also similar to those reported in previous reports [1,2,8]. In addition, we further evaluated the NIPS performance for common trisomies among different risk cohorts. PPVs of Trisomy 21 demonstrated a higher accuracy among cohorts with ultrasound soft markers (95%), DSS high-risk result (94.81%) and AMA (92.82%) cohorts, as comparable to other risk cohorts (ranged 75–85%). Our data also supported the ACOG to advocate the use of NIPS for common aneuploidies in all pregnancies regardless of any risk, especially for high-risk population (higher PPV), and demonstrated the robustness of the NIPS platforms even in less developed autonomous region. Our study not only enhanced the accuracy of evaluating the FNR for each type of chromosomal abnormalities by NIPS, but it also provided a baseline of birth defects/adverse pregnancies (2.92%, 2405/82,511) in less developed autonomous region. The data demonstrated that most of NIPS negative cases with birth defects (99.83%, 2401/2405) were not affected by the common chromosomal abnormalities.

We also compared the combined PPV for SCAs (38.00%), which was also comparable to that reported by expanded NIPS (46.7%) [8]. Overall, the PPV (12.04%) for monosomy X was lower than previously reported from two sequencing platforms (19.39% to 28.57%) [3]. One of the reasons might be that the PPV in the AMA cohort was extremely low (4.29%) compared to the other groups (Table 4). However, we cannot exclude the low PPV contributed by confined placental mosaicism [28] or maternal monosomy X mosaicism [29]. PPVs for SCAs reported by NIPS (lower PPV compared with the one for common trisomies) are extremely important for the pregnant women’s decision-making. For cases with suspected Monosomy X, apart from reporting a simple Monosomy X, complex chromosomal abnormalities involving X chromosome such as translocations, isochromosomes, rings were identified in eight cases by further invasive testing. Observation of such complex rearrangements was similar to the previous study [30]. For instance, among the 179 cases with high-risk result by NIPS but did not pursue invasive confirmation; 82.9% (87/105) of cases reported to have SCAs that opted for continuing pregnancies, significantly higher than that with confirmation of SCAs (47.4%, 72/152, *p* < 0.001, Table A4). Another reason might be that most of the fetuses with SCAs except monosomy X would have less lethality, as compared to the ones with common trisomies. 

In our study, we also explored the applicability of standard NIPS in identifying other chromosomal abnormalities. First, standard NIPS was able to provide a 33.3% PPV for reporting CNVs (>5 Mb), which was also comparable to the one reported by expanded NIPS (40.8%). However, the number of reported CNV cases (13/86,193) was much less than the one reported by expanded NIPS in a cohort with similar sample size (163/94,085) [8]. Despite low FPR for these extra abnormalities, the relatively low PPVs obtained might lead to increase the risks of parental anxiety and the unnecessary invasive procedure. In addition, previous prenatal CMA findings indicated that 84.0% of known pathogenic CNVs are less than 5 Mb [31], which were unlikely to be detectable even by expanded NIPS. For the segmental chromosomal abnormalities, fetuses would commonly present with ultrasound anomalies, which warrants an invasive test for comprehensive genetic evaluation via CMA or genome sequencing (low-pass or high read-depth) [22,32,33]. 

In addition, our study also showed low-to-moderate PPVs for RATs (20.45%), which were also likely comparable to those reported by expanded NIPS (6 to 28.6%) [8,10]. For Multis, confound factors such as maternal tumor [34] and systemic lupus erythematosus [35] were known, while two cases with maternal blood disease and gallstone were found in our study (2/18 with further follow-up). In the context that current guidelines on reporting such events (CNVs and RATs) are still controversial [36]. As in China, Guangxi is an economically underdeveloped area, with limited resources (including limited support in new technology development; lack of cost-effective prenatal testing, such as NIPS, available; and absence of technical staff supports). Although expanded NIPS have been developed internationally, the application is restricted by the limited resources. In addition, the charge fee of standard NIPS (USD221 per case), including USD60 as reagent cost, is comparable to the routine G-banded chromosome analysis (USD230 per case). Therefore, standard NIPS is still regarded as an affordable prenatal screening test for common aneuploidies and SCAs in the Guangxi region.

Moreover, among 10,541 pregnancies with high-risk results by DSS (mainly enriched by DSS2 cohort), only 1.9% (201/10,541) of samples yielded high-risk results by NIPS, which can potentially prevent about 98% pregnant women from obtaining a further invasive prenatal diagnosis. Similarly, in previous study [1], NIPS yielded an extremely lower FPR (nearly 100 times) and higher PPV (80.9%) for common trisomies, as compared with standard screening (with measurement of nuchal translucency and biochemical analytes, 3.4%). Therefore, the performance of standard NIPS from a less developed autonomous region in Mainland China is superior to DSS methods, with reporting lower FPR and higher PPV. In additional, standard NIPS yielded high sensitivity (>90.48%) and specificity (>99%) in detecting common trisomies and SCAs (XXY, XXY, XYY) among different referral indications; however, relatively lower sensitivity of trisomy 18 in ultrasound soft markers group (*n* = 4, sensitivity = 75%) and monosomy X in low-risk cohort (*n* = 33, no clinical indications group, sensitivity = 75%) were observed. While the PPVs for trisomy 21 (75–95%), SCAs (XXY, XXY, XYY, 47–78%) and monosomy X (0–50%) were variable, there were no significant differences in these referral indications (*p* > 0.05) (Table 4). Therefore, the results demonstrated the use of standard NIPS detection of common trisomies and SCAs is feasibility in any risk cohorts, especially when there were no other screening options available in this less developed region. Overall, the information will be useful for clinical counseling, to help and support pregnant women in the Guangxi region to go for NIPS.

The strengths of our study include (1) a large sample size and outcome follow-up data (*n* = 83,671, 97.07%) from (2) a single center and local cohort in a less developed region and (3) consecutive pregnancies from a prospective cohort. However, there are limitations we need to face: (1) There was a lack of samples with expanded NIPS for comparison in study, as it is expensive and not allowed for a large-sample size application. (2) Despite low FPR for these abnormalities, relatively low PPVs (such as Monosomy X, CNVs and RATs) were still existed and might lead to increase the risks of parental anxiety and the unnecessary invasive procedure. (3) Almost all false negative cases were confirmed by karyotyping/SNP microarray; however, there were still a large number of cases with abnormal phenotypes (such as abnormal ultrasound, birth defects, stillbirth and miscarriage) lacking further genetic diagnosis requesting for follow-up genetic diagnosis with advanced methods. (4) Because chromosomal abnormalities are known to cause variable phenotype presentations, especially for a phenotype with atypical abnormality and intellectual disability, which is difficult to identify during the pregnancy, they could lead to the loss of a follow-up. This raises important issues regarding genetic counseling, which is underdeveloped in Guangxi. (5) Lastly, the majority of our cases was from the second-trimester pregnancy (*n* = 78,231, 90.76%), while there was only 0.68% of cases from the third-trimester pregnancy, which is relatively small (*n* = 584, 0.68%, cf-DNA = ~23%), preventing a proper comparison; therefore, a larger sample size of cases from the third-trimester pregnancy is warranted for comparison in the near future.

In order to improve clinical management for pregnant women choosing NIPS, there are efforts we must take: entirely complete monitoring information (ultrasonography, postnatal CT and MRI) and establish workflow and management for prenatal diagnosis and follow-up, especially improving abilities for specialized counseling in local region. Since completed diagnosis and follow-up data can be recorded and collected from a specific system in Guangxi, this, in turn, will promote clinical practice and further study of NIPS in local regions. 

## 5. Conclusions

In summary, our study with sufficient follow-up data (97.07%, 83,671/86,193), demonstrated that standard NIPS can provide a sensitive screening method in the detection of fetal common aneuploidies and sex chromosomal abnormalities. Standard NIPS may be a cost-effective method for routine prenatal screening in a less developed region in Mainland China.

## Figures and Tables

**Table 1:** Characteristics of the pregnant women with NIPS results in our study.

	N	Mean ± SD	Median	Range
Age (years)	86,193	32.89 ± 5.59	33.83	16–58.00
16–24	8099	22.21 ± 2.13	22.75	16–24.92
25–29	18,353	27.71 ± 1.39	27.83	25–29.92
30–34	22,354	32.51 ± 1.48	32.50	30–34.92
35–39	30,618	37.13 ± 1.38	37.00	35–39.92
40–44	6528	41.53 ± 1.20	41.25	40–44.92
≥45	241	46.39 ± 1.59	45.92	45–58.00
GA at sampling (weeks)	86,193	17^+5^ ± 3 W	17^+3^ W	12–38 W
First-trimester	7378	12^+2^ W ± 1 W	13^+1^ W	12–13^+6^ W
Second-trimester	78,231	17^+5^ W ± 3 W	17^+1^ W	14–27^+6^ W
Third-trimester	584	29^+1^ W ± 2 W	29 W	28–38 W
Maternal BMI (kg/m^2^)	82,695	22.30 ± 3.10	22.00	13.32–41.42
<18.5	7177	17.59 ±0.77	17.78	13.32–18.49
18.5–24.9	61,157	21.68 ± 1.72	21.64	18.50–24.99
25.0–29.9	12,905	26.71 ± 1.28	26.44	25.00–29.99
30.0–34.9	1324	31.61 ± 1.31	31.25	30.00–34.96
35.0–39.9	123	36.91 ± 1.42	36.63	35.00–39.96
≥40	9	40.86 ± 0.44	40.79	40.10–41.42
Mode of conception				
Spontaneous	72,753(84.41%)			
ART	2802(3.25%)			
Unknown	10,638(12.34%)			

NIPS, noninvasive prenatal screening; BMI, body mass index; GA, gestational age; ART, assisted reproductive technology.

**Table 2:** Information of detection failure on the originally submitted and resubmitted samples.

No.	Failure Reasons	Blood/Plasma Samples ^1^			DNA/Library ^2^		Sequencing Data Analysis ^3^					
		A	B	C	D	E	F	G	H	I	J	K
1.	Original samples											
1.1.	Experiment failure cases (first tube plasma)	204	222	20	132	1145	65	152	85	133	1077	22
	Experiment failure rate% (first tube plasma, total cases)	0.52 (446/86,262)			1.48(1277/86,262)		1.78(1534/86,262)					
1.1.1.	Re-experiment rate% (second or third tube plasma, re-examining)	/			1.48(1277/86,262)		1.58 (1359/86,262)					
1.1.2.	Re-experiment failure rate%	/			0.06(48/86,262)		0.15(128/86,262)					
2.	Resampling procedure											
2.1.	Resampling rate% (required resampling)	0.52 (446/86,262)			0.06(48/86,262) ^4^		0.35(303/86,262) ^5^					
2.2.	Actual resampling rate% (accepted and obtained)	0.52 (446/86,262)			0.03(22/86,262)		0.32 (279/86,262)					
2.3.	Experiment failure rate% (redrawing samples)	/			0(0/22)		6.81(19/279)					
2.4.	Experiment successful rate% (redrawing samples)	100(446/446)			100(22/22)		93.19(260/279)					
3.	Final failure rate%											
3.1.	Final test failure rate% (no results, original and resampling)	/			0.03 ((48–22)/86,262)		0.03 ((303–279) + 19)/86,262) ^6^					
	Final test failure cases (total cases) ^7^	0.08(69/86,262)										
4.	Average fetal fraction% (accepted and obtained)	*n* = 125										
4.1.	Result group ^8^						12.61 (5.49%, *n* = 110)					
4.2.	No-call group ^8^						10.59 (2.82%, *n* = 15)					

^1^ Poor quality of whole blood or plasma: A, hemolysis; B, coagulation; C, others, e.g., lipemia, hemolysis with coagulation. ^2^ DNA/library issues: D, high/low concentration of DNA extraction; E, abnormal peak, low concentration of library construction. ^3^ Sequencing data issues: F, GC content bias (>42 or <38); G, insufficient data amount; H, multiple chromosome abnormalities; I, low fetal fraction (FF), FF < 3.0%; J, borderline Z-score; K, multiple factors, failed samples with more than one failing factor, including high GC, insufficient data amount and low fetal fraction, multiple chromosome abnormalities, etc. ^4^ These samples received a non-reportable NIPS results even after re-experiment with the second or third plasma of the original samples. ^5^ In total, 0.35% of cases required resampling totally. Among these cases, 0.15% (*n* = 128) still failed in the re-experiment with the second or third plasma of the original samples, and 0.2% (*n* = 175) were required for resampling directly, mainly due to low FF (*n* = 133). ^6^ A total of 0.03% failure cases were included 24 re-experiment cases and 19 resampling cases. ^7^ All of these failure cases chose to terminate the test. ^8^ Result/no-call groups: defined as with/without results yielded from the direct resampling due to low FF (*n* = 125).

**Table 3:** Performance of NIPS in screening of chromosome abnormalities in 83,671 general pregnancies ^1^.

No.	NIPS Result	N	TP (*n*)	FP (*n*)	UC (*n*)	FN (*n*)	Sensitivity (%)	Specificity (%)	PPV (%)	NPV (%)	FPR (%)	FNR (%)	SPR (%)
Part 1: Common aneuploidies													
1.	Trisomy 21	368	330	38	51	3	99.1	99.95	89.67	99.996	0.05	0.9	0.5
	Trisomy 21(full)	349	325	24	50	3	99.09	99.97	93.12	99.996	0.03	0.91	
	Trisomy 21(mos)	19	5	14	1	0	100	99.98	26.32	100	0.02	0	
2.	Trisomy 18	100	84	16	8	1	98.82	99.98	84	99.999	0.02	1.18	0.13
	Trisomy 18	95	83	12	8	1	98.81	99.99	87.37	99.999	0.01	1.19	
	Trisomy 18(mos)	5	1	4	0	0	100	100	20	100	0	0	
3.	Trisomy 13	57	30	27	2	0	100	99.97	52.63	100	0.03	0	0.07
	Trisomy 13	50	29	21	2	0	100	99.97	58	100	0.03	0	
	Trisomy 13(mos)	7	1	6	0	0	100	99.99	14.29	100	0.01	0	
4.	Common aneuploidies (combined)	525	444	81	61	4	99.11	99.9	84.57	99.995	0.1	0.89	0.7
Part 2: SCAs													
1.	Monosomy X	191	23	168	48	3	88.46	99.8	12.04	99.996	0.2	11.54	0.29
	Monosomy X	185	23	162	46	3	88.46	99.81	12.43	99.996	0.19	11.54	
	Monosomy X (mat)	6	0	6	2	0	/	99.99	0	100	0.01	/	
2.	Other SCAs (XXX, XXY, XYY, combined)	184	128	56	52	0	100	99.93	69.57	100	0.07	0	0.28
	XXX	53	36	17	18	0	100	99.98	67.92	100	0.02	0	0.08
	XXY	113	78	35	29	0	100	99.96	69.03	100	0.04	0	0.17
	XYY	18	14	4	5	0	100	100	77.78	100	0	0	0.03
3.	46,XY(delX)	25	1	24	5	0	100	99.97	4	100	0.03	0	0.04
4.	SCAs (combined)	400	152	248	105	3	98.06	99.7	38	99.996	0.3	1.94	0.6
Part 3: Other abnormalities													
1.	CNVs (≥5 Mb) ^2^	12	4	8	1	16	20	99.99	33.33	99.981	0.01	80	0.02
2.	RATs ^3^	44	9	35	12	1	90	99.96	20.45	99.999	0.04	10	0.07
3.	Other abnormalities (combined)	56	13	43	13	17^4^	43.33	99.95	23.21	99.98	0.05	56.67	0.09
Part 4: Common aneuploidies, SCAs and other abnormalities													
1.	Common aneuploidies and SCAs (combined)	925	596	329	166	7	98.84	99.6	64.43	99.992	0.4	1.16	1.3
2.	Totally (combined)	981	609	372	179	24	96.21	99.55	62.08	99.971	0.45	3.79	1.39

NIPS, noninvasive prenatal screening; N, invasive diagnosis and clinical diagnosis; TP, true positive; FP, false positive; UC, refused to diagnosis; FN, false negative; PPV, positive predictive value; NPV, negative predictive value; FPR, false positive rate; FNR, false negative rate; SPR, screen-positive rate; Mos, mosaic. ^1^ A total of 83,671 pregnancies, included 1160 screening positive cases and 82,511 negative cases with follow-up data. ^2^ CNVs, copy number variants (≥5 Mb), these CNVs included fragments sizes of chromosome deletion and duplication ranged from 6 to 32.5 Mb and 5.5 to 99 Mb, respectively (Table A2). ^3^ RATs, rare autosomal aneuploidies (chrN+/-), including, increased dosage chromosomes (most likely trisomies, chrN+), decreased dosage chromosomes (most likely monosomies, chrN-) and multiple chromosomal abnormalities (Multis, more than two chromosomal abnormalities) (Table A2 and Table A3). Almost all the Multis were retested again. ^4^ A total of 17 false negative cases were all confirmed by SNP microarray.

**Table 4:** Performance and classifications of NIPS in different risks pregnancy cohort groups ^1^.

No.	NIPS Result Classifications of Different-Risk Population	N	TP (*n*)	FP (*n*)	FN (*n*)	UC (*n*)	Sensitivity (%)	Specificity (%)	PPV (%)	NPV (%)	FPR (%)	FNR (%)	SPR (%)
Cohort 1	AMA ^2^ populations (≥35 years, N = 36,491), high-risk by NIPS (*n* = 595)												1.63
	Trisomy 21	209	194	15	2	36	98.98	99.96	92.82	99.99	0.04	1.02	0.67
	Trisomy 18	57	50	7	0	10	100	99.98	87.72	100	0.02	0	0.18
	Trisomy 13	33	17	16	0	7	100	99.96	51.52	100	0.04	0	0.11
	Monosomy X	70	3	67	0	14	100	99.82	4.29	100	0.18	0	0.23
	Other SCAs (XXX, XXY, XYY)	98	77	21	0	19	100	99.94	78.57	100	0.06	0	0.32
	46,XY(delX)	14	0	14	0	1	/	99.96	0	100	0.04	/	0.04
	CNVs(≥5 Mb)	5	2	3	8	1	20	99.99	40.00	99.98	0.01	80	0.02
	RATs	18	5	13	0	3	100	99.96	27.78	100.00	0.04	0	0.06
	CNVs/RATs (combined)	23	7	16	8	4	46.67	99.96	30.43	99.98	0.04	53.33	0.07
Cohort 2	DSS ^3^ high-risk populations (T21 ≥ 1/270, T18 ≥ 1/350), N = 10,541, high-risk by NIPS (*n* = 201)												1.91
	Trisomy 21	77	73	4	0	14	100	99.96	94.81	100	0.04	0	0.86
	Trisomy 18	16	13	3	1	4	92.86	99.97	81.25	99.99	0.03	7.14	0.19
	Trisomy 13	5	3	2	0	0	100	99.98	60	100	0.02	0	0.05
	Monosomy X	30	7	23	0	14	100	99.78	23.33	100	0.22	0	0.42
	Other SCAs (XXX, XXY, XYY)	22	12	10	0	5	100	99.9	54.55	100	0.1	0	0.26
	46,XY (delX)	4	1	3	0	2	100	99.97	25	100	0.03	0	0.06
	CNVs (≥5 Mb)	1	1	0	4	0	20	100	100	99.96	0	80	0.01
	RATs	5	0	5	0	2	/	99.95	0	100	0.05	/	0.07
	CNVs/RATs (combined)	6	1	5	4	2	20	99.95	16.67	99.96	0.05	80	0.08
Cohort 3	DSS ^3^ intermediate-risk populations (1/1000 ≤ T21 ≤ 1/270 or 1/1000 ≤ T18 ≤ 1/350, N = 14,375), high-risk by NIPS (*n* = 168)												1.17
	Trisomy 21	34	29	5	0	8	100	99.97	85.29	100	0.03	0	0.29
	Trisomy 18	8	7	1	0	1	100	99.99	87.5	100	0.01	0	0.06
	Trisomy 13	3	0	3	0	0	/	99.98	0	100	0.02	/	0.02
	Monosomy X	48	7	41	1	9	87.5	99.71	14.58	99.99	0.29	12.5	0.4
	Other SCAs (XXX, XXY, XYY)	23	11	12	0	13	100	99.92	47.83	100	0.08	0	0.25
	46,XY (delX)	1	0	1	0	2	/	99.99	0	100	0.01	/	0.02
	CNVs (≥5 Mb)	3	0	3	3	0	0	99.98	0	99.98	0.02	100	0.02
	RATs	10	3	7	0	5	100	99.95	30	100	0.05	0	0.10
	CNVs/RATs (combined)	13	3	10	3	5	50	99.93	23.08	99.98	0.07	50	0.12
Cohort 4	Ultrasound soft markers populations (N = 1786), high-risk by NIPS (*n* = 38)												2.13
	Trisomy 21	20	19	1	2	4	90.48	99.94	95	99.89	0.06	9.52	1.34
	Trisomy 18	3	3	0	1	0	75	100	100	99.94	0	25	0.17
	Trisomy 13	1	1	0	0	0	100	100	100	100	0	0	0.06
	Monosomy X	2	1	1	0	2	100	99.94	50	100	0.06	0	0.22
	Other SCAs (XXX, XXY, XYY)	3	2	1	0	1	100	99.94	66.67	100	0.06	0	0.22
	46,XY (delX)	0	0	0	0	0	/	100	/	100	0	/	0
	CNVs (≥5 Mb)	0	0	0	1	0	0	100	/	99.94	0	100	0
	RATs	2	0	2	1	0	0	99.89	0	99.94	0.11	100	0.12
	CNVs/RATs (combined)	2	0	2	2	0	0	99.89	0	99.89	0.11	100	0.12
Cohort 5	Maternal anxieties populations ^4^ (N = 52,292), high-risk by NIPS (*n* = 660)												1.26
	Trisomy 21	210	185	25	1	35	99.46	99.95	88.1	100	0.05	0.54	0.47
	Trisomy 18	52	44	8	1	10	97.78	99.98	84.62	100	0.02	2.22	0.12
	Trisomy 13	34	16	18	0	7	100	99.97	47.06	100	0.03	0	0.08
	Monosomy X	98	11	87	2	29	84.62	99.83	11.22	100	0.17	15.38	0.24
	Other SCAs (XXX, XXY, XYY)	107	74	33	0	24	100	99.94	69.16	100	0.06	0	0.25
	46,XY (delX)	16	0	16	0	4	/	99.97	0	100	0.03	/	0.04
	CNVs (≥5 Mb)	2	1	1	11	1	8.33	100	50	99.98	0	91.67	0.01
	RATs	26	5	21	0	5	100	99.96	19.23	100	0.04	0	0.06
	CNVs/RATs (combined)	28	6	22	11	6	35.29	99.96	21.43	99.98	0.04	64.71	0.07
Cohort 6	ART populations ^5^ (N = 2730), high-risk by NIPS (*n* = 37)												1.36
	Trisomy 21	15	13	2	1	1	92.86	99.93	86.67	99.96	0.07	7.14	0.59
	Trisomy 18	4	4	0	0	0	100	100	100	100	0	0	0.15
	Trisomy 13	2	0	2	0	0	/	99.93	0	100	0.07	/	0.07
	Monosomy X	6	0	6	0	2	/	99.78	0	100	0.22	/	0.29
	Other SCAs (XXX, XXY, XYY)	5	3	2	0	2	100	99.93	60	100	0.07	0	0.26
	46,XY (delX)	0	0	0	0	0	/	100	/	100	0	/	0
	CNVs (≥5 Mb)	0	0	0	2	0	0	100	/	99.93	0	100	0
	RATs	0	0	0	0	0	/	100	/	100	0	/	0
	CNVs/RATs (combined)	0	0	0	2	0	0	100	/	99.93	0	100	0
Cohort 7	Others ^6^, (N = 11,914), high-risk by NIPS (*n* = 104)												0.87
	Trisomy 21	20	15	5	1	2	93.75	99.96	75	99.99	0.04	6.25	0.18
	Trisomy 18	9	5	4	0	1	100	99.97	55.56	100	0.03	0	0.08
	Trisomy 13	2	1	1	0	0	100	99.99	50	100	0.01	0	0.02
	Monosomy X	28	3	25	1	4	75	99.79	10.71	99.99	0.21	25	0.27
	Other SCAs (XXX, XXY, XYY)	19	13	6	0	10	100	99.95	68.42	100	0.05	0	0.24
	46,XY(delX)	3	0	3	0	0	/	99.97	0	100	0.03	/	0.03
	CNVs (≥5 Mb)	3	1	2	1	0	50	99.98	33.33	99.99	0.02	50	0.03
	RATs	2	0	2	0	1	/	99.98	0	100	0.02	/	0.03
	CNVs/RATs (combined)	5	1	4	1	1	50	99.97	20	99.99	0.03	50	0.06

NIPS, noninvasive prenatal screening; N, invasive diagnosis and clinical diagnosis; TP, true positive; FP, false positive; UC, refused to diagnosis; FN, false negative; PPV, positive predictive value; NPV, negative predictive value; FPR, false positive rate; FNR, false negative rate; SPR, screen-positive rate. SCAs, sex chromosome abnormalities; CNVs, copy number variants (≥5 Mb); RATs, rare autosomal aneuploidies. ^1^ Some cases may be counted repeatedly in different categories. The samples without follow-up results were excluded. ^2^ AMA, advanced maternal age. ^3^ DSS, these cohorts included pregnancy women who have undergone prenatal screening either in the first-trimester (DSS1) or second-trimester (DSS2), or those who have done both of these screenings before NIPS. However, these cohorts were mainly enriched by DSS2 populations. ^4^ Maternal anxieties mainly include (1) histories of pregnancy loss or recurrent miscarriage or termination of pregnancy, or congenital malformation; (2) contacted with chemical teratogens and X-ray, or suffer from diseases; (3) threatened abortion in current pregnancy. ^5^ ART, assisted reproductive technology. ^6.^ Others, no clinical indications.

## Data Availability

Data available on request due to the ethical restrictions.

## Appendix A

**Table A1:** The relationship between Z score and fetal fraction in common trisomies by NIPS.

Common Trisomies	Basic Information (All)						Basic Information of Male-Population (cf-DNA% Calculated by Y)								
	Z Score	*n*	NIPS-TP		NIPS-FP		*n*	NIPS-TP				NIPS-FP			
			*n* (Ratio %)	Mean Z Score	*n* (Ratio %)	Mean Z Score		*n* (Ratio %)	Mean Z Score	Mean FF% (Range)	*p*-Value	*n* (Ratio %)	Mean Z Score	Mean FF% (Range)	*p* Value
Trisomy 21	3 ≤ Z < 6	41	15 (36.59)	4.57	26 (63.41)	4.63	23	11 (47.83)	4.66	8.29 (4.01–13.03)	<0.001	12 (52.17)	4.83	10.01 (4.37–16.11)	<0.05
	6 ≤ Z < 10	79	70 (88.61)	7.98	9 (11.39)	7.55	51	45 (88.24)	7.95	10.4 (6.05–33.04)		6 (11.76)	7.65	14.26 (7.32–22.00)	
	10 ≤ Z < 16	149	147 (98.66)	12.93	2 (1.34)	14.45	79	79 (100.00)	12.85	14.42 (8.52–22.36)		0 (0.00)	―	―	
	Z ≤ 16	90	90 (100.00)	21.28	0	―	44	44 (100.00)	21.06	21.26 (10.55–34.86)		0	―	―	
	Total	359	322		37		197	179				18			
Trisomy 18	3 ≤ Z < 6	26	12 (46.15)	4.69	14 (53.85)	4.14	10	5 (50.00)	4.22	7.41 (3.27–15.86)	<0.002	5 (50.00)	4.54	11.95 (6.73–14.94)	
	6 ≤ Z < 10	27	26 (96.30)	8.12	1 (3.70)	6.27	12	11 (91.67)	8.09	8.72 (4.61–16.58)		1 (8.33)	6.27	6.9	/
	10 ≤ Z < 16	34	33 (97.06)	12.57	1 (2.94)	11.52	14	14 (100.00)	12.21	11.95 (8.71–22.47)		0 (0.00)	―	―	
	Z ≥ 16	13	13 (100.00)	20.9	0	―	7	7 (100.00)	20.07	15.94 (12.52–22.17)		0	―	―	
	Total	100	84		16		43	37				6			
Trisomy 13	3 ≤ Z < 6	23	7 (30.43)	4.91	16 (69.57)	4.44	8	2 (25.00)	4.53	6.95 (3.10–10.80)	=0.143	6 (75.00)	4.84	13.78 (7.27–22.19)	=0.869
	6 ≤ Z < 10	21	12 (57.14)	7.76	9 (42.86)	7.19	11	7 (63.64)	7.72	8.53 (5.14–12.75)		4 (36.36)	6.6	14.35 (10.58–20.59)	
	10 ≤ Z < 16	11	10 (90.91)	12.11	1 (9.09)	10.57	7	7 (100.00)	11.82	11.24 (6.96–16.47)		0 (0.00)	―	―	
	Z ≥ 16	2	1 (50.00)	16.66	1 (50.00)	17.14	1	0 (0.00)	―	―		1 (100.00)	17.14	22.6	
	Total	57	30		27		27	16				11			

NIPS, noninvasive prenatal screening; TP, true positive; FP, false positive; FF, fetal fraction.

**Table A2:** Detailed diagnostic information of NIPS–CNVs/RATs results and follow-up study.

No.	GA	NIPS Results	NIPS-Sizes	Karyotyping-Fetus	SNP Microarray- Fetus	TP/ FP	Follow-up	Maternal Diseases
Part 1: CNVs (≥5 Mb)								
1.	19	chr21-	chr21:15500000_22999999_loss_mat/fetal (7.5 Mb); chr21:23500000_29499999_gain_fetal (6 Mb); chr21:32000000_47999999_loss_fetal (16 Mb)	46,XN	arr(1-22)x2,(XN)x1	FP	Liveborn/normal	―
2.	17	chr21-	chr21:15500000_47999999_loss_fetal (32.5 Mb)	46,XN	arr(1-22)x2,(XN)x1	FP	Liveborn/normal	―
3.	19	chr18-	chr18:63000000_77999999_loss_fetal (15 Mb)	46,XN	arr10p15.3p14(135708-8975723)x3; arr18q22.1q23(63490350-78014582)x1, pathogenic	TP	TOP/limb anomalies	―
4.	18	chr18-	chr18:50000000_77999999_loss_fetal (28 Mb)	46,XN,del(18)(q21)[45]/46,XN [5]	arr 18q21.2q23(53260732-77931598)x1, pathogenic	TP	TOP	―
5.	16	chr18-	chr18:0_6999999_loss_mat/fetal (7 Mb)	46,XN,del(18)(p11.3)	arr 18p11.31p11.32(12842-6759762)x1, pathogenic	TP	Liveborn/normal	―
6.	19	chr18-	chr18:55500000_77999999_loss_fetal (22.5 Mb); chr8:135000000_146499999_gain_mat/fetal (11.5 Mb)	46,XN,der(18)t(8,18)(q24,q21)	arr 8q24.2q24.3(135140240-146293086)x3; arr 18q21.3q23(55448382-78014582)x1, pathogenic	TP	TOP	―
7.	17	chr13-	chr13:19500000_32999999_gain_mat/fetal (13.5 Mb); chr13:91500000_114999999_loss_fetal (23.5 Mb)	46,XN	―	FP	Liveborn/normal	―
8.	24	chr13-	chr13:55000000_71499999_gain_fetal (16 Mb); chr13:85000000_90999999_loss_mat/fetal (6 Mb)	46,XN	―	FP	Liveborn/normal	―
9.	17	chr4+	chr4:500000_8999999_gain_fetal (8.5 Mb);	―	―	―	Miscarriage	―
10.	16	chr14+	chr14:20500000_107499999_gain_mat/fetal (87 Mb);	46,XN	arr(1-22)x2,(XN)x1	FP	Liveborn/normal	―
11.	17	chr7+	chr7:0_56999999_gain_fetal (57 Mb); chr7:65500000_158999999_gain_fetal (93.5 Mb)	46,XN	―	FP	Liveborn/normal	―
12.	18	chr8+	chr8:12500000_42499999_gain_fetal (30 Mb); chr8:47500000_146499999_gain_fetal (99 Mb)	46,XN	arr(1-22)x2,(XN)x1	FP	Liveborn/normal	―
13.	20	chr9+	chr9:0_5499999_gain_mat/fetal (5.5 Mb)	46,XN	arrXp22.32p22.31(5919409-8452482)x3, variants of uncertain significance	FP	Liveborn/normal	―
Part 2: RATs								
1.	17	chr21-	―	46,XN	―	FP	Liveborn/normal	―
2.	16	chr21-	―	46,XN	arr(1-22)x2,(XN)x1	FP	Liveborn/normal	―
3.	19	chr21-	―	46,XN	arr(1-22)x2,(XN)x1	FP	Liveborn/normal	―
4.	17	chr21-	―	46,XN	arr21q22.3(44769969-46720010)x1, variants of uncertain significance	TP	Liveborn/normal	―
5.	19	chr18-	―	46,der(18)dup(18)(q11q22)del(18)(q22q23)	arr18q11.2q22.2(24719986-68043316)x3; arr18q22.2q23(68050903-78014582)x1, pathogenic	TP	TOP/polyhydramnios	―
6.	16	chr18-	―	―	―	―	Liveborn/normal	―
7.	18	chr18-	―	46,XN	arr18q23(74,231,996-78,014,582)x1; arr18q23(74227095-77129563)x1, like pathogenic	TP	Liveborn/refuse	―
8.	15	chr18-	―	46,XN	arr18q22.1q22.2(63959330-67189360)x1, like pathogenic	TP	Liveborn/preterm labor, low weigh, small head circumference	―
9.	17	chr18-	―	46,XN	arr(1-22)x2,(XN)x1	FP	Liveborn/normal	―
10.	18	chr18-	―	―	―	―	Liveborn/normal	―
11.	20	chr13-	―	46,XN	―	FP	Liveborn/normal	―
12.	16	chr13-	―	46,XN	arr(1-22)x2,(XN)x1	FP	Liveborn/normal	―
13.	21	chr13-	―	―	―	―	Liveborn/normal	―
14.	15	chr1+	―	46,XN	arr(1-22)x2,(XN)x1	FP	Liveborn/normal	―
15.	16	chr22+	―	46,XN	arr(1-22)x2,(XN)x1	FP	Liveborn/normal	―
16.	17	chr7+	―	46,XN	―	FP	Liveborn/normal	―
17.	18	chr14+	―	―	―	―	Liveborn/normal	―
18.	17	chr17+	―	―	―	―	Liveborn/normal	―
19.	15	chr9+	―	46,XN	arr(1-22)x2,(XN)x1	FP	Liveborn/normal	―
20.	18	chr14-	―	46,XN	arr(1-22)x2,(XN)x1	FP	Liveborn/normal	―
21.	16	chr7+	―	46,XN	arr(1-22)x2,(XN)x1	FP	Liveborn/normal	―
22.	18	chr8+	―	―	―	―	Liveborn/normal	―
23.	21	chr7+	―	46,XN	arr16p13.12p13.3(110925-13904865)x2,hmz, variants of uncertain significance	FP	Liveborn/normal	―
24.	16	chr3+	―	46,XN	arr(1-22)x2,(XN)x1	FP	Liveborn/normal	―
25.	19	chr20+	―	―	―	―	Liveborn/normal	―
26.	17	chr1+	―	―	―	―	Liveborn/normal	―
27.	20	chr7+	―	―	―	―	TOP	―
28.	18	chr16-	―	―	―	―	Liveborn/normal	―
29.	14	chr8+	―	46,XN	arr(1-22)x2,(XN)x1	FP	Liveborn/normal	―
30.	13	chr11+	―	46,XN	arr(1-22)x2,(XN)x1	FP	Liveborn/normal	―
31.	12	chr7+	―	46,XN	arr(1-22)x2,(XN)x1	FP	Liveborn/normal	―
32.	30	chr8+	―	46,XN	arr(1-22)x2,(XN)x1	FP	Liveborn/normal	―
Part 2: RATs (Multis)								
33.	16	T21(mos),T13(Mos)	―	46,XN	arr(1-22)x2,(XN)x1	FP	Liveborn/normal	Normal
34.	20	T21,chrX-	―	47,XN,+21	―	TP	TOP	Normal
35.	16	T21,46,XY(delX)	―	46,XN	arr(1-22)x2,(XN)x1	FP	Liveborn/normal	Normal
36.	22	T21,T18,T13,chr1+,chr2+,chr3+,chr4+,chr5+,chr6+,chr7-,chr8+,chr9+,chr12+,chr14+,chr16+,chr20+	―	46,XN	―	FP	Liveborn/normal	Aplastic anemia/Chinese herbal treatment
37.	17	T21(mos),T13(mos),chr4+	―	46,XN	arr(1-22)x2,(XN)x1	FP	Liveborn/normal	Normal
38.	18	T21,XXX,chr1+/−,chr12-,chr13-	chr1:21000000_56499999_loss_fetal (35.5 Mb); chr1:152500000_183999999_gain_fetal (31.5 Mb); chr1:198500000_223999999_gain_fetal (25.5 Mb); chr1:224500000_248999999_loss_fetal (24.5 Mb); chr12:43000000_49499999_loss_fetal (6.5Mb); chr12:73500000_81999999_loss_fetal (8.5 Mb); chr12:87500000_92999999_loss_fetal (5.5 Mb); chr13:19500000_55499999_loss_fetal (35 Mb); chr13:94500000_114999999_loss_fetal (20.5 Mb)	47,XN,+21	arr(21)x3	TP	TOP/abnormal US (no exact details)	Normal
39.	18	T21(mos),chr1+/−,chr2+,chr4-,chr5-,chr6+,chr7+,chr8-,chr9+,chr10-,chr12+,chr13-,chr16-,chr19+/−	chr1:15000000_120499999_loss_fetal (105.5 Mb); chr1:150000000_248499999_gain_fetal (98.5 Mb); chr4:0_26999999_loss_fetal (27 Mb); chr6:158000000_170999999_gain_fetal (13 Mb); chr8:0_32499999_loss_fetal (24.5 Mb); chr9:1000000_34999999_gain_fetal (34 Mb); chr10:1000000_38499999_loss_fetal (37.5 Mb); chr10:54000000_123999999_loss_fetal (70 Mb); chr19:500000_24499999_gain_mat/fetal (24 Mb); chr19:28000000_58999999_loss_fetal (31 Mb)	46,XN	arr(1-22)x2,(XN)x1	FP	Liveborn/normal	Normal
40.	16	T18,T13,chr5+/−,chr6+,chr8+,chr9+,chr11+,chr14+,chr17-,chr20+	chr5:17500000_46499999_gain_fetal (16 Mb); chr5:49500000_180499999_loss_mat/fetal (131 Mb); chr17:0_7499999_loss_mat/fetal (7.5 Mb)	46,XN	arr(1-22)x2,(XN)x1	FP	Liveborn/normal	―
41.	17	T18,XXY	―	48,XXY,+18	arr(18)x3,(X)x2,(Y)x1	TP	TOP	―
42.	20	T18, XXY	―	46,XN	arr(1-22)x2,(XN)x1	FP	Liveborn/normal	Normal
43.	24	T18,T13,chr1-,chr2+,chr5+,chr17-	―	46,XN	arr(1-22)x2,(XN)x1	FP	Liveborn/normal	Gallstone
44.	20	T18,T13,chr21-	―	46,XN	arr(1-22)x2,(XN)x1	FP	Liveborn/normal	―
45.	18	T18(mos),T13(mos),chr4+,chr5+,chr8+,chr15+,chr17+	―	46,XN	arr(1-22)x2,(XN)x1	FP	Liveborn/normal	Normal
46.	17	T18, chr1+,chr2,chr7+, chr8+,chr9,chr10-,chr14-,chr16-	―	46,XN,1qh+,t(8;12)(q11;q21)	arr18q12.1q21.2(30255638-48336327)x2,hmz, variants of uncertain significance	TP	TOP	―
47.	16	T18,chrX+	―	―	―	―	Miscarriage	―
48.	16	T13,chrX-,chr1-,chr2-,chr3-,chr4-,chr5-,chr6+,chr7-,chr8+,chr9-,chr10+,chr11-,chr12-,chr14+,chr16-,chr17+,chr18-,chr20+,chr21-,chr22+	―	46,XN	―	FP	Liveborn/normal	―
49.	20	T13mos,chr6+,chr15-,chr16-,chr17-,chr20-,chr22-	―	46,XN	arr(1-22)x2,(XN)x1	FP	Liveborn/normal	Normal
50.	16	T13,chrX+,chr1-,chr8+,chr17-,chr22-	―	46,XN	―	FP	Liveborn/normal	Normal
51.	24	46,XY(delX),chr13-,chr4-,chr8-	―	―	―	―	Liveborn/normal	Normal
52.	17	chr8-,chr17+,chr18-,chr19+	―	46,XN	arr(1-22)x2,(XN)x1	FP	Liveborn/normal	Normal
53.	12	chrX-,chr4-,chr13-	―	46,XN	arr 16q23.1q23.2(78,173,154-79,354,550)x3, variants of uncertain significance	FP	Liveborn/normal	Normal
54.	21	chrX-,chr2-,chr3-,chr6-,chr7-,chr8+,chr9-,chr10-,chr11-,chr12-,chr13-,chr18-, chr21-	―	46,XN	arr(1-22)x2,(XN)x1	FP	Liveborn/normal	Normal
55.	18	chr1-,chr7+,chr10-,chr17-, chr18-	chr7:0_56999999_gain_fetal (57 Mb); chr7:65500000_158999999_gain_fetal (93.5 Mb)	46,XN	arr(1-22)x2,(XN)x1	FP	Liveborn/normal	Normal
56.	21	chr2-,chr4-,chr5+,chr6-,chr17-,chr20-,chr21-	chr4:134800000_190799999_loss_fetal (56 Mb); chr5:87000000_102199999_gain_fetal (15.2 Mb); chr6:22000000_29499999_loss_fetal (7.5 Mb); chr17:10200000_21299999_loss_fetal (11.1 Mb); chr20:5300000_16099999_loss_fetal (10.8 Mb)	46,XN	arr5q14.2q15(82201161-94331486)x2,hmz, variants of uncertain significance	TP	Liveborn/normal	Normal

NIPS, noninvasive prenatal screening; TP, true positive; FP, false positive; GA, gestational age; CNVs, copy number variants; RATs, rare autosomal aneuploidies; Multis, multiple chromosome abnormalities; TOP, termination of pregnancy; US, ultrasound.

**Table A3:** Prenatal diagnosis for NIPS false negative cases and follow-up study.

No.	NIPS- Results	GA	Karyotyping-Fetus	SNP Microarray-Fetus	Size(Mb)				PC ^1^	Category ^2^	Pregnancy Outcome ^3^	Phenotypes
Part 1: Twenty-four cases with common trisomies, SCAs and CNVs (≥5 Mb) and RATs were confirmed (*n* = 24)												
1.	Low-risk	16	46,der(21;21)(q10;q10),+21	arr(21)x3					P	(1) FN	Adverse	fetal duodenal atresia
2.	Low-risk	22	47,XN,+21	unconfirm					P	(1) FN	Liveborn	congenital heart disease, respiratory infections and special face
3.	Low-risk	12	47,XN,+21	unconfirm					P	(1) FN	Liveborn	fetal polyhydramnios, special face
4.	Low-risk	12	47,XN,+18	arr(18)x3					P	(1) FN	Adverse	fetal congenital heart disease, increased nuchal fold, overlapping hand, hygroma
5.	Low-risk	18	unconfirm	arr(X)x1~2					P	(2) FN	Adverse	― ^4^
6.	Low-risk	16	unconfirm	arr(X)x1~2					P	(2) FN	Adverse	―
7.	Low-risk	14	failed	arr(Y)x0~1					P	(2) FN	Adverse	fetal enlargement of posterior fossa
8.	Low-risk	14	unconfirm	arr16q13.13(12323566-12551122)x3; arr16p13.12(13662679-14219837)x3; arr18p11.32p11.31(12842-6588865)x1	0.23	0.56	6.58		P	(3) FN	Adverse	fetal renal dysplasia, polyhydramnios
9.	Low-risk	17	47,XN,1qh+,+der(22)t(11;22)(q23;q11)	arr11q23.3q25(116728277-134944006)x3; arr22q11.1q11.21(16079545-20305944)x3	18.22	4.23			P	(3) FN	Adverse	fetal dysgenesis of the corpus callosum, ventriculomegaly
10.	Low-risk	13	46,XN,del(6)(q14.3q22.1)	arr6q14.3q22.1(87977775-115590427)x1	27.61				P	(3) FN	Adverse	fetal congenital heart disease
11.	Low-risk	15	46,XN	arr6q25.1q27(151602119-1645553480x2 hmz	1493.95				VOUS	(3) FN	Liveborn	fetal arrhythmia, normal neonate/infancy period
12.	Low-risk	16	46,XN	arr5q11.2q12.1(56368573-61428613)x1	5.06				LP	(3) FN	Liveborn	fetal cardiac abnormality congenital heart disease
13.	Low-risk	19	46,XN	arr2q11.1q11.2(95537501-98658823)x2,hmz; arr16q21q23.2(79092703-90148796)x2,hmz	3.12	11.06			VOUS	(3) FN	Liveborn	fetal cerebral ventriculomegaly, normal neonate/infancy period
14.	Low-risk	13	unconfirm	arrq22.31q23.1(119,443,290-130,985,191)x2,hmz	11.54				VOUS	(3) FN	Liveborn	fetal choroid plexus cysts normal neonate/infancy period
15.	Low-risk	14	46,XN	arr2q31.1q32.1(171500167-187802572)x2, hmz	16.30				VOUS	(3) FN	Liveborn	normal neonate/infancy period
16.	Low-risk	17		arr11p15.5p15.3(204228-10760363)x2,hmz	10.56				LP	(3) FN	Liveborn	normal neonate/infancy period
17.	Low-risk	17	46,XN	arr14q32.13q32.33(95423213-105463936)x2, hmz	10.04				LP	(3) FN	Adverse	―
18.	Low-risk	16	unconfirm	arr10q26.3(131642219-135430043)x2,hmz; 13q14.11q21.33(43207878-71161023)x2,hmz; arr17p11.2(18851012-22175355)x2,hmz; arr17q11.1q12(25402163-34272942)x2,hmz	3.79	27.95	3.32	8.87	VOUS	(3) FN	Liveborn	normal neonate/infancy period
19.	Low-risk	17	46,XN	arr20q11.21q13.12(29846402-45461021)x2,hmz	15.61				VOUS	(3) FN	Adverse	fetal congenital heart disease
20.	Low-risk	16	unconfirm	arr14q21.3q24.1(49351716-70190601)x2,hmz	20.84				VOUS	(3) FN	Liveborn	fetal renal dysplasia, ectopic kidney
21.	Low-risk	22	unconfirm	arr2p13.3p11.2(69173570-84392155)x2,hmz	15.22				VOUS	(3) FN	Liveborn/low weight	fetal hydronephrosis, atresia of anus and rectum, developmental delay, short stature
22.	Low-risk	16	unconfirm	arr7q21.11q22.2(82214954-104112216)x2; arr8p12p11.21(35424457-41204995)x2; arr17q25.3(76142795-79173184)x2	21.90	5.78	3.03		VOUS	(3) FN	Liveborn	normal childhood
23.	Low-risk	18	46,XN	arr7p22.3p22.1(62643-6054987)x3; arr18q23(77336836-78014582)x1	5.99	0.68			P	(3) FN	Adverse	fetal separation of renal pelvis
24.	Low-risk	30	47,XX,+22[2]/46,XX[48]	arr(22)x2~3					P	(4) FN	Liveborn	congenital heart disease, polydactyly, deafness
Part 2: Twenty-nine cases with CNVs (<5 Mb, lower detection limit of NIPS) were confirmed (*n* = 29)												
25.	Low-risk	12	46,XN	arr13q31.3(93506864-94240082)x3; arr16p11.2(29634212-30192561)x1	0.73	0.56			P	(5)	Adverse	renal agenesis
26.	Low-risk	17	46,XX	arr16p11.2(29656093-30328317)x1	0.67				P	(5)	Liveborn	renal agenesis
27.	Low-risk	37	46,XY	arr6q16.3(103467436-103669065)x1	0.20				LB	(5)	Liveborn	normal neonate/infancy period
28.	Low-risk	14	46,XX	arrXp22.31 (6516735-8131442)x3	1.61				VOUS	(5)	Adverse	―
29.	Low-risk	14	46,XY	arr16p13.11(15126890-16289532)x3	1.16				VOUS	(5)	Liveborn	normal neonate/infancy period
30.	Low-risk	14	46,XY	arr21q21.2(24936629-26661518)x1	1.72				VOUS	(5)	Adverse	neck neoplasm
31.	Low-risk	17	46,XX	arr16p13.3(216516-271712)x0	0.06				P	(5)	Adverse	―
32.	Low-risk	16	46,XY	arr22q11.21(18,877,787-21,798,907)x1	2.92				P	(5)	Adverse	congenital heart disease
33.	Low-risk	12	unconfirm	arr16p13.3(217411-257548)x0	0.04				P	(5)	Adverse	fetal thickening of nuchal translucency
34.	Low-risk	15	46,XY	arr22q11.21(20740778-21445064)x1	0.70				P	(5)	Liveborn	congenital heart disease
35.	Low-risk	17	46,XY	arr10p12.31(19026841-21207377)x3	2.18				VOUS	(5)	Liveborn	fetal hydronephrosis, normal neonate/infancy period
36.	Low-risk	18	unconfirm	arr1q41(222991420-223276713)x1	0.29				VOUS	(5)	Liveborn	fetal single umbilical artery, normal neonate/infancy period
37.	Low-risk	20	unconfirm	arr4q34.3(178160858-179872123)x3	1.71				VOUS	(5)	Adverse	fetal ventricular bright spot, cardiac abnormality
38.	Low-risk	17	46,XX,1qh+	arr5q35.3(177410416-180554812)x2-3	3.14				VOUS	(5)	Liveborn	fetal left ventricular bright spot, hyperechogenic bowel, normal neonate/infancy period
39.	Low-risk	19	46,XX	arr17p11.2(16705818-18775900)x1	2.07				P	(5)	Adverse	Fetal cerebral ventriculomegaly
40.	Low-risk	17	46,XY	arr6q26(162236682-162799117)x1	0.56				VOUS	(5)	Liveborn	normal neonate/infancy period
41.	Low-risk	16	unconfirm	arr3p21.1(52880740-54205850)x3	1.33				VOUS	(5)	Adverse	fetal congenital heart disease
42.	Low-risk	17	46,XY	arr22q11.21(18889490-21462353)x1	2.57				P	(5)	Liveborn	congenital heart disease
43.	Low-risk	14	unconfirm	arr5q23.1q23.2(119816120-121644484)x3	1.83				VOUS	(5)	Liveborn /low weight	normal neonate/infancy period
44.	Low-risk	16	46,XY	arr16p13.3(216050-286982)x0	0.07				P	(5)	Adverse	fetal severe thalassemia and hydrops
45.	Low-risk	18	46,XY	arr8p22(15743626-16882361)x3	1.14				VOUS	(5)	Adverse	fetal cleft lip and palate
46.	Low-risk	20	unconfirm	arrYp11.31p11.2(2878213-6616258)x2	3.74				VOUS	(5)	Adverse	―
47.	Low-risk	17	46,XY	arr6q16.3(103467436-103669065)x1	0.20				LB	(5)	Liveborn	fetal left ventricular bright spot, normal neonate/infancy period
48.	Low-risk	23	46,XX	arr17q12(34815551-36249565)x1	1.43				P	(5)	Adverse	fetal polycystic kidney
49.	Low-risk	15	unconfirm	arr16p13.3(230,578-381,927)x0	0.15				P	(5)	Adverse	fetal cardiac abnormality, severe thalassemia and hydrops
50.	Low-risk	15	46,XX	arr1q21.1q21.2(146501348-148349952)x3	1.85				P	(5)	Adverse	―
51.	Low-risk	17	unconfirm	arr16p13.3(216738-420907)x0	0.20				P	(5)	Adverse	fetal hydrops
52.	Low-risk	17	46,XX	arr15q11.2q13.1(23683301-28544359)x1	4.86				P	(5)	Adverse	fetal single umbilical artery
53.	Low-risk	18	failed	arr11p14.3(23337714-24690002)x1	1.35				VOUS	(5)	Adverse	fetal cardiac abnormality, hydrops

NIPS, noninvasive prenatal screening; TP, true positive; FP, false positive; GA, gestational age; SCAs, sex chromosome abnormalities; CNVs, copy number variants (≥5 Mb); RATs, rare autosomal aneuploidies. ^1^ PC, pathogenic classification. P, pathogenic; LP, like pathogenic; VOUS, variants of uncertain significance; LB, like benign. ^2^ The chromosome polymorphism variations (diagnosed by karyotype) were not included in the statistics. (1) FN, T21/T18/T13; (2) FN, Monosomy X; (3) FN, CNVs ≥ 5 Mb; (4) FN, RAT; (5) CNVs < 5 Mb (lower detection limit of NIPS). ^3^ Adverse, including stillbirth, miscarriage and opted to termination of pregnancy. ^4^―, Lost follow-up.

**Table A4:** Follow-up study in high- and low-risk results of NIPS.

Part 1: High-risk of NIPS (cases)			
Ⅰ. Basic information			
ⅰ. Total high-risk results	1160		
ⅱ. Refused genetic diagnosis	179		
ⅲ. Diagnosis (Karyotyping/SNP microarray/clinical phenotype) Diagnosis rate (T21, T18 and T13) Diagnosis rate (SCAs)	981 87.83%, 92.59%, 96.61% 79.21%		
Ⅱ. confirmed and pregnancy outcomes	**Common trisomies (T21, T18, T13)**	**SCAs**	**Other abnormalities**
ⅰ. True positive (N = 609)			
Liveborn	22 (9 cases were clinical diagnosis with Down syndrome)	72	5
Terminate the pregnancy/Adverse	422	80	8
ⅱ. False positive (N = 372)			
Liveborn	81	247	43
Terminate the pregnancy/Adverse	0	1	0
ⅲ. Refused to diagnosis (N = 179)			
Liveborn	14 (Normal live birth)	87	10
Terminate the pregnancy/adverse	47	18	3
Part 2: Low-risk results of NIPS (cases)			
Ⅰ. Basic information	Liveborn	Death of fetal (stillbirth/miscarriage/termination of pregnancy)	Lost to follow-up
Total low-risk 85,033 (86,193-1160)	81,348 (95.67%)	1163 (1.37%)	2522 (2.96%)
Ⅱ. Live-birth	Spontaneous labor	Caesarean birth	
ⅰ. Delivery mode (N = 81,342)	52,018 (63.95%)	29,330 (36.05%)	
ⅱ. Delivery gestation weeks (mean ± SD)	38.61 W ± 1.55 W		
ⅲ. Sex ratio,	Men/Women = 1.07		
ⅳ. Birth weight(kg) (mean ± SD)	3175.41 ± 637.12 (All)	3224.18 ± 597.32 (Men)	3120.18 ± 603.75 (Women)
Ⅲ. Phenotype (live birth)	80,106 (98.47%) Normal	1242 (1.53%) Birth defects	
The detailed information for birth defects are as followed (1242 cases, 1.51% (1242/82,511)),			
developmental delay, metabolic disorders			537
skeletal and limb deformities (e.g., cleft lip and palate, polydactyly, spina bifida and talipes equinovarus)			236
congenital heart disease			117
ear deformity, dysaudias			114
congenital urogenital malformations (e.g., renal agenesis, polycystic and ectopic kidney, pyelic separation, hypospadias and micropenis)			77
hydrocephalus, brain paralysis, abnormal brain development			19
alimentary tract malformation (e.g., bowel atresia, Hirschsprung, biliary atresia and vascular malformation)			15
multi-system, multi-malformations (e.g., congenital heart disease, duodenal stenosis, respiratory infections, special face, cleft lip and palate, dysaudia and polydactyly)			7
others (e.g., tumors, hemangiomas, hernia, patent foramen ovale and other defects)			120
Ⅳ. Stillbirth/miscarriage/termination of pregnancy (cases)			
ⅰ. Total	1163 (1.41%, 1163/82,511)		
ⅱ. Risk factors and causes of fetal death (630 cases),			
(1) Maternal factors			
hypertension, eclampsia, preeclampsia, diabetes, hypothyroidism, liver/heart/renal disease, fibroids, autoimmune disease, drugs, etc.			41
cervical insufficiency, uterine rupture, etc.			41
severe anemia, malnutrition, maternal trauma or death, etc.			22
history of abortion			12
(2) Placental factors			
premature rupture of membranes			54
cord accident (e.g., umbilical cord entrapment and fetal single umbilical artery)			33
pathologic placental conditions (e.g., previa, accrete and velamentous)			18
(3) Maternal, fetal, and/or placental factors			
fetomaternal hemorrhage, infections, etc.			13
(4) Fetal factors			
congenital heart disease, fetal cardiac abnormality			91
cleft lip and palate, spina bifida, polydactyly/limb deformities, talipes equinovarus, skeletal dysplasia, etc.			55
polyhydramnios, anhydramnios, hydrops, intrauterine growth restriction, etc.			29
renal disease (e.g., renal agenesis, polycystic and ectopic kidney and hydronephrosis)			23
lung disease (e.g., pulmonary stenosis and pulmonary hypoplasia)			17
anencephaly, hydrocephalus, encephalocele, dysgenesis of the corpus callosum, abnormal brain development			16
multiple and/or multisystem malformations (e.g., congenital heart disease, facial abnormalities, cerebellar hypoplasia, anencephaly, hydrocephalus, agenesis of corpus callosum, increased nuchal fold, overlapping hands, polydactyly, spina bifida, hygroma, renal agenesis and polyhydramnios)			35
other factors (e.g., duodenal atresia, eye and ear diseases, adenoma/tumor or genetic abnormality)			60
(5) Family/personal factors (e.g., accidents and poverty)			70
ⅲ. Not clear	533		
Ⅴ. Diagnostic follow-up of NIPS in low-risk (cases)			
ⅰ. Liveborn (744 cases)	Total	normal	abnormal
chromosome karyotyping	490	473	17
SNP microarray	661	638	23
Total diagnosis (no double counting) ^1^	744	707	37
Karyotyping/SNP microarray, phenotyping (aneuploidy); 47,XX,+21, congenital heart disease, respiratory infections and special face; 47,XX,+21, polyhydramnios and special face			
ⅱ. Stillbirth/Miscarriage/termination of pregnancy	Total	normal	abnormal
chromosome karyotyping	63	59	4
SNP microarray	89	61	28
total diagnosis (no double counting)	95	66	29
Karyotyping/SNP microarray, phenotyping (aneuploidy); arr(21)x3/46,der(21;21)(q10;q10),+21 and duodenal atresia; 47,XX,+18/arr(18)x3 and congenital heart disease, increased nuchal fold, overlapping hand, hygroma			

NIPS, noninvasive prenatal screening; SCAs, sex chromosome abnormalities. ^1^ Including chromosome polymorphism (13 cases)
